# Exploring the relationship between self-efficacy, social support, academic anxiety, and academic outcomes: a meta-analysis structural equation modeling approach

**DOI:** 10.3389/fpsyg.2025.1714845

**Published:** 2025-12-10

**Authors:** Yun Wang, Dongyun Li, Juntao Li, Shengnan Bai

**Affiliations:** 1 School of Mathematics and Statistics, Henan Normal University, Xinxiang, China; 2 School of Mathematics and Statistics, Henan University, Kaifeng, China

**Keywords:** academic anxiety, academic outcomes, meta-analysis, self-efficacy, social support

## Abstract

**Goal:**

This study examined the correlation between self-efficacy, social support, academic anxiety, and academic outcomes. A structural equation model was constructed with academic outcomes as the dependent variable and self-efficacy as the independent variable. Social support and academic anxiety are introduced as mediating variables.

**Methods:**

A total of 59 studies published between July 2001 and February 2025 were obtained from the Web of Science, EBSCO, Taylor and Francis, Scopus, Wiley, ProQuest, and CNKI (core) databases, comprising 95 independent samples (total sample size = 49,072).

**Results and discussion:**

The results showed that self-efficacy was positively correlated with academic outcomes at a moderate to high level (*r* = 0.354), social support was positively correlated with academic outcomes at a moderate to low level (*r* = 0.245), and academic anxiety was negatively correlated with academic outcomes at a moderate to low level (*r* = −0.179). All correlations between variables were significant (*p* < 0.001). Cultural background, grade level and the types of academic outcomes measurement exerted moderating effects on some relationships: under Eastern culture, the influence of social support on academic outcomes was stronger, and the influence of self-efficacy on academic outcomes increased with grade level. The relationship between self-efficacy and academic outcomes was stronger in the objective measures group. Structural equation modeling confirmed that social support and academic anxiety mediated the relationship between self-efficacy and academic outcomes, with a total indirect effect of 14.12%. This study identified how self-efficacy, social support, and academic anxiety shape academic outcomes, providing practical insights for enhancing student learning.

## 1 Introduction

Academic outcomes (AO) are not only important variables for cognitive ability, positive psychological functioning and educational system performance (Organisation for Economic Co-operation and Development, 2017), but are also moderately to strongly correlated with an individual’s future socio-economic status (Sirin, 2005). According to the results of the Program for International Student Assessment (PISA), countries with high test scores in all subjects generally perform better in the Global Competitiveness Index (GCI). Research shows mathematics, science, and reading scores, as well as higher education enrollment rates, all correlate significantly positively with the GCI. Countries or regions with higher PISA test scores, such as Singapore, Finland, South Korea, and Japan, also rank at the forefront in global competitiveness rankings (Alexander, 2012). UNSECO 2030 lists “ensuring inclusive and equitable quality education” as a core objective, requiring that coverage and quality of education be monitored through assessments of AO. Therefore, it is important to explore the key factors and mechanisms that influence AO for both individual development and national competitiveness (United Nations Educational, Scientific and Cultural Organization, 2015).

As the primary agents of the learning process, students’ intrinsic psychological factors such as self-efficacy (SE) and academic anxiety (AA) influence AO. Based on data from British students, Putwain et al. (2013) found that SE plays a significant role in maintaining positive emotions and achieving better learning outcomes, with an effect size of 0.21. Further research by Du et al. (2020) on Chinese students revealed an even stronger correlation between SE and AO, with a correlation coefficient of 0.452. Most existing studies suggest that math anxiety negatively predicts students’ math performance (Du et al., 2020; Hembree, 1990; Ma, 1999). However, data from Italy indicates a weak positive correlation between AA and AO (Möcklinghoff et al., 2023). Previous study found that mathematics anxiety plays a complete mediating role between SE and AO. In addition to internal psychological factors, students’ perception of social support (SS), which including teacher support, peer support, and parental support also influences their AO (Kenyatta University and Masisi, 2024; Virtanen et al., 2020). When faced difficulties, students with abundant SS tend to adopt problem-solving-oriented positive coping strategies, thereby significantly enhancing their AO. Recent evidence has shown that SS plays an important role in mitigating adolescents’ psychological difficulties, suggesting its broader relevance for understanding AA in student populations (Alshehri et al., 2020). In summary, early research has identified associations among SE, SS, AA, and AO. However, inconsistencies exist across studies regarding the interrelationships and effects of SE, SS and AA on AO, and the precise mechanisms underlying these relationships remain unclear.

Due to the considerable heterogeneity and inconsistency observed across existing research findings, it remains difficult to draw robust universal conclusions regarding the strength and stability of the relationships among variables based on individual studies. This limitation further obstructs a comprehensive understanding of potential moderating mechanisms. As a key methodological approach for integrating quantitative evidence, meta-analysis allows for the systematic synthesis and reanalysis of results from a large number of independent studies. By transcending the constraints inherent in single-study designs, it facilitates a more precise quantification of overall effect sizes and helps pinpoint sources of between-study heterogeneity (Lee, 2015). Within the current research context, the application of meta-analysis is essential to reconcile conflicting results, elucidate potential moderating variables, and contribute to a consensus-based knowledge framework on the mechanisms underlying AO. Accordingly, this study employed a meta-analytic approach, incorporating 95 independent samples to synthesize prior research in line with predefined criteria. Furthermore, we sought to investigate potential interrelationships among these variables.

## 2 Literature review and research hypotheses

### 2.1 Self-efficacy

SE refers to an individual’s belief and assessment of their ability to successfully engage in specific behaviors. Individuals with high SE tend to set higher goals and exert greater effort to achieve them. It is a core element that directly or indirectly promotes academic performance 2004. The correlation between SE and AO has been confirmed. Putwain et al. (2013) found that SE plays a significant role in maintaining positive emotions and achieving better AO. SE is also closely linked to SS and AA. Lei et al. (2022) concluded that academic SE is significantly positively correlated with SS, meaning that students with higher academic SE perceive higher levels of SS. Research has also shown that self-efficacy is closely linked to individuals’ emotional and psychological functioning, such as its predictive role in loneliness during the COVID-19 pandemic (Yıldırım et al., 2022). When faced with exam anxiety or academic setbacks, students with high SE are more likely to view stress as motivation and maintain focus through positive self-talk. In contrast, those with low SE are more prone to falling into negative emotions (Pajares, 1996). Though the correlation between SE and AO is well established, researchers are not unanimous about the extent of the correlation between the two. Li et al. (2024) found that higher SE led to better AO, with correlation coefficients of up to 0.47, by administering questionnaires and tests to 483 junior high school students at three different points in a semester. However, Wang et al. (2024) concluded that the correlation between geography SE and geography grades was only 0.09. Therefore, current research findings are inconsistent, necessitating the use of meta-analysis methods to synthesize existing research results.

### 2.2 Social support

SS refers to the respect, care, and assistance perceived by the recipient from significant others or other groups (Pierce et al., 1996). The ecological perspective recognizes that students are significantly influenced by their social surroundings (Bronfenbrenner, 1986). This opinion provides a way to understand the relationship between SS and AO (Dennis et al., 2005). SS provides students with a sense of security and competence, which in turn helps them to cope more effectively with intellectual challenges (Sarason et al., 1990). Recent studies further demonstrate that social support may influence outcomes through complex pathways, including indirect effects via psychosocial mechanisms such as social exclusion (Koçak et al., 2025). Numerous relevant studies provided empirical support for the relationship between AO and SS. For example, students’ successful academic functioning relies heavily on the interpersonal support they receive from their teachers and peers (Burns et al., 2019; Tao et al., 2022). A previous meta-analysis, however, found only weak positive correlations between perceived SS and its subtypes and AO (Wu et al., 2023). It was further noted that neither parental, teacher nor peer support was a statistically significant direct predictor of AO, implying that the general hypothesis about the importance of perceived SS in AO may be overestimated (Kristensen et al., 2023). As a result, there has not been a clear and definitive conclusion about the extent to which SS correlates with AO.

### 2.3 Academic anxiety

AA refers to the negative emotional state experienced by students during academic activities, encompassing feelings of tension, unease, fear, and anxiety toward academic tasks and the learning process. Early research indicates that AA is prevalent among adolescents and is closely linked to AO and mental and physical health. Moderate AA can help stimulate students’ motivation to learn and maintain their focus in the classroom. However, prolonged severe AA can have a negative impact on students’ AO (Du et al., 2020). Although most researchers believe that there is a negative correlation between AA and AO, there are also studies that show some degree of positive correlation. One study found a positive correlation of 0.06 between AA and AO through a cross-sectional study that included 746 Italian students (Möcklinghoff et al., 2023). It has also been noted that students with higher SE are better able to regulate their learning activities and academic emotions, and therefore show less anxiety, stress, or frustration. On the other hand, students with lower SE show anxiety, uneasiness or irrational fear due to lack of competence, avoid activities related to exercises and problem solving, and even slack off in class or drop out of school, which in turn affects AO (Yüksel and Geban, 2015). Owing to these conflicting findings, further research is warranted.

### 2.4 Academic outcomes

Academic outcomes can be measured by test scores or academic grades. The more specific the definition and measurement of learning outcomes, the more likely it is to identify the effects of causal relationships (Cohen, 1987). For example, scores in specific subjects are more preferable than grade point averages, and more effective than aptitude test scores, self-reported AO, and AO grades reported by teachers. This study included in this meta-analysis uniformly used school test scores or subject outcomes test scores to measure AO.

### 2.5 Relevant moderating variables

In a number of meta-analysis studies, the individual’s stage of internal development and the external cultural context were identified as the primary moderating variables. The primary moderator is the grade or age stage of the adolescent. According to Erikson’s (1968) theory of psychosocial development, adolescents in different school grades are at different stages of psychosocial crisis. The effects of their SE, perceived SS, and AA on AO will vary. For example, as they grow older, the significance of AO transcends mere proof of competence and becomes important fodder for constructing and validating self-identity (Erikson, 1968). As a result, the role of SE increases significantly. Another important moderating dimension is the external cultural context. Western cultures are more individualistic, while Eastern cultures are more collectivist (Markus and Kitayama, 1991). In collectivist cultures, individuals are more likely to view themselves as interdependent with the group, leading them to place greater emphasis on meeting collective expectations, be more susceptible to others’ influence, and feel greater anxiety about failing to meet collective expectations compared to individuals in individualistic societies. AO are measured in various ways, including standardized test scores, course grades, teacher ratings, and student self-reports. Objective indicators tend to more accurately reflect students’ actual abilities, yielding more stable relationships with psychological variables. By contrast, subjective evaluations can be influenced by rater standards, students’ psychological states, or social expectations, potentially amplifying or attenuating these relationships. Therefore, treating the type of AO measurement as a moderator can help explain the heterogeneity in effect sizes across studies. As a key external resource shaping students’ AO, SS has also long been recognized for its diversity and heterogeneity. Existing research has demonstrated that parental support, teacher support, and peer support—three core types of SS among adolescents—exert distinct influences on AO (Dupont et al., 2015). Therefore, this study further differentiates SS into these three dimensions and incorporates them into a moderator framework. Based on this, the present study incorporates cultural background, grade level, the types of AO measurement, and the types of SS into the meta-analytic model to systematically examine the relationships of academic AA, SS, and AA with AO, as well as their potential moderating effects.

In summary, current research on the factors influencing students’ AO is not uniform. One of the main reasons for this is that most of the existing studies focus on a single variable or a two-by-two relationship between variables, failing to reveal the potential dynamic interactions between SE, SS and AA, as well as the overall mechanism by which they work together to affect AO, making it difficult to clearly present the real impact of the factors on AO and the paths of their effects. The meta-analysis approach can make up for these shortcomings. By integrating the results of multiple independent studies, it is able to increase the sample size and improve the statistical testing power, thus estimating the strength of the relationship between variables more precisely. At the same time, meta-analysis can systematically examine the heterogeneity among different studies, identify moderating variables that may affect the results of the study, and provide more reliable and comprehensive evidence for clarifying the influencing factors and pathways of students’ AO. Therefore, the present study used 95 independent samples (including 49,072 student samples) from 59 domestic and international papers to calculate the weighted correlation coefficients between the variables using meta-analysis, constructed the correlation coefficient matrix, and then examined the pathways of academic SE affecting students’ AO through SS and AA using structural equation modeling (SEM), taking into account the moderating influences of cultural background and academic level. The moderating influence of the academic year.

On the basis of this, this paper proposes the following research hypotheses:

*H1*: SE, SS and AA will affect AO to some extent.

*H2*: Cultural background, grade level, the types of AO measurement, and the types of SS served as moderators in the relationships among the variables.

*H3*: SS and AA mediate between SE and AO.

## 3 Methods

### 3.1 Literature search strategy

**Figure 1:**
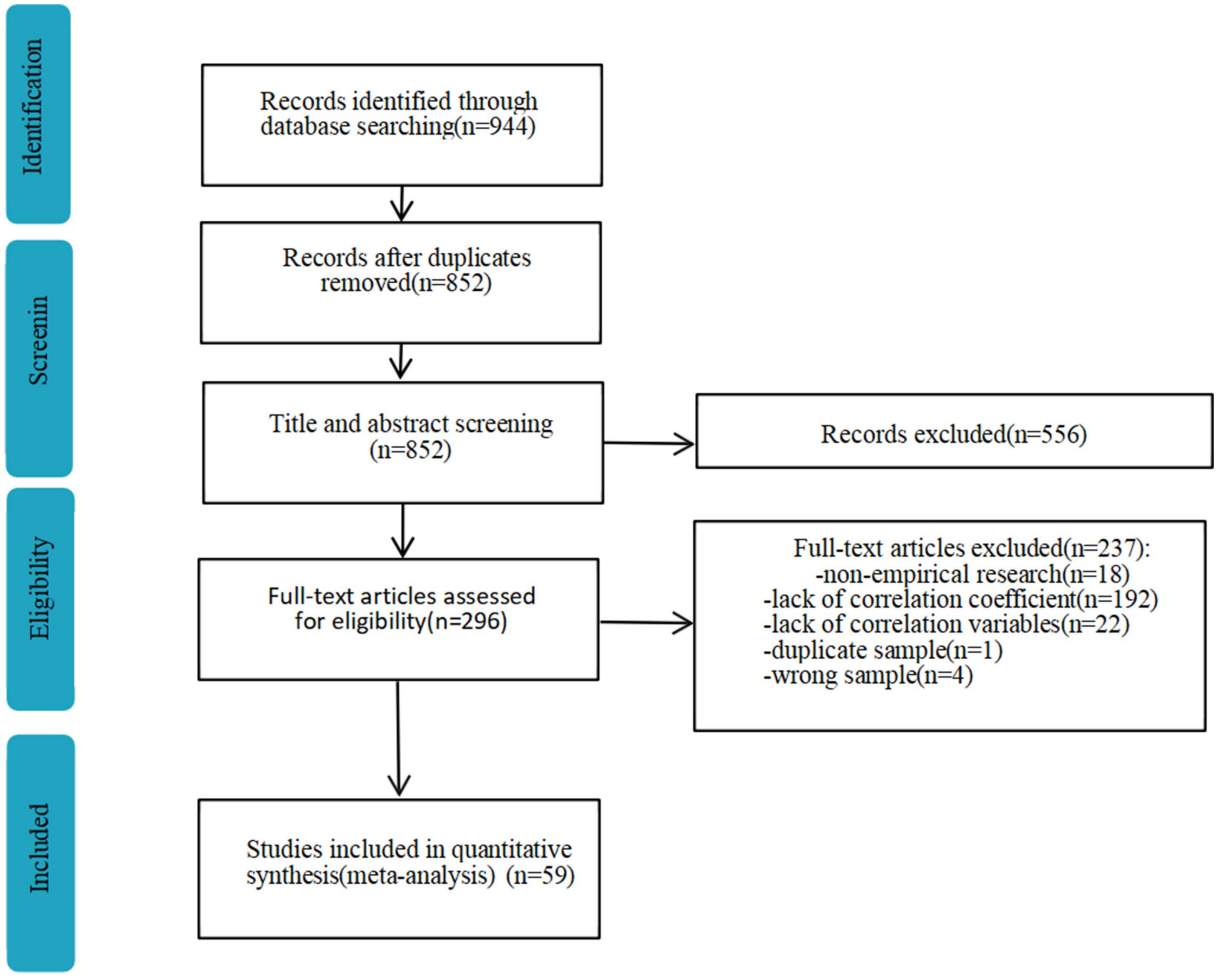
Flowchart of the study selection process.

Web of Science, EBSCO, Taylor and Francis, Scopus, Wiley, ProQuest and CNKI (core) databases were systematically searched. Since the international interest in monitoring and large-scale assessment of educational quality increased significantly around 2001, providing a sufficient set of data sources for meta-analyses, English-language journal articles and dissertations, as well as Chinese-language journal articles published between July 2001 and February 2025, were searched for this paper. A total of 1,766 studies were retrieved (Figure 1).

### 3.2 Criteria for literature screening

Inclusion criteria are as follows: (1) published empirical research journal articles or theses; (2) covering the predictor variable (SE), two mediators (SS and arbitrary subgroups: family [primarily parents], peers [classmates and friends], and school [school and teachers] support; AA); (3) Literature reporting sample size and correlation coefficients r or other indicators of convertible data; (4) Literature including adolescents from primary school to university, excluding preschool-aged groups; (5) If the literature uses the same set of data, the group with the most accurate data is considered; (6) Cohort studies using baseline data, excluding literature where baseline data cannot be extracted.

### 3.3 Variable coding

The studies included in this meta-analysis were coded for multiple characteristics, including the first author and publication year, sample size, relevant correlations, educational level, cultural context, the types of AO measurement, the types of SS and gender ration (male/female). Educational level was categorized into primary, secondary, and higher education. Cultural background was coded as Eastern or Western culture. Eastern culture was limited to the East Asian cultural sphere, while Western culture encompassed countries and regions in Europe, North America, and Oceania. Standardized test scores and course grades were coded as objective measures, whereas teacher ratings and student self-reports were coded as subjective evaluations. When studies report correlation coefficients for specific subtypes of SS, the variable will be coded as Parental Social Support (PSS), Teacher Social Support (TSS), and Peer Social Support (PeSS). If a study includes two or more independent samples that meet the criteria, the samples are coded separately. Two researchers independently extracted information and completed the coding process. In cases of disagreement or inconsistency, a third researcher was involved to reach consensus. The basic characteristics of the included literature are shown in Table 1.

**Table 1:** Characteristics of the included studies.

**Study name**	***N***	**Grade level**	**Culture**	**Outcomes measure**	**Publication**	**Variable relation**	**Gender ratio**
Lu et al. (2022)	498	College	Eastern	Objective measures	Journal	SE&AO	0.22
Xing and Dou (2021)	1,072	Middle school	Eastern	Subjective evaluations	Journal	SE&AO	0.78
Cai and Yang (2019)	901	College	Eastern	Objective measures	Journal	SE&AO	0.59
Guo et al. (2017)	379	Primary school	Eastern	Subjective evaluations	Journal	SE&AO	1.26
Wei et al. (2014)	407	Primary school	Eastern	Subjective evaluations	Journal	SE&AO	1.08
Du et al. (2020)	787	Middle school	Eastern	Subjective evaluations	Journal	SE&AA&AO	1.04
Du et al. (2020)	787	Middle school	Eastern	Subjective evaluations	Journal	SE&AA&AO	1.04
Chen (2022)	614	Middle school	Eastern	Subjective evaluations	Journal	SS&AO	0.81
Zhao et al. (2018)	1,328	Middle school	Eastern	Objective measures	Journal	SS&AO	0.63
Chen and Guo (2016)	1,379	Middle school	Eastern	subjective evaluations	Journal	TSS&AO	1.15
Guo and Bian (2013)	1,330	Middle school	Eastern	Objective measures	Journal	PeSS&AO	male only
Guo and Bian (2013)	1,137	Middle school	Eastern	Objective measures	Journal	PeSS&AO	female only
Xiao and Liu (2017)	7,089	Primary school	Eastern	Subjective evaluations	Journal	PSS&AO	1.09
Gan (2024)	2,079	Middle school	Eastern	Subjective evaluations	Journal	SE&AA&AO	0.96
Gan (2024)	2,079	Middle school	Eastern	Subjective evaluations	Journal	SE&AA&AO	0.96
Lin and Yi (2022)	1,423	Middle school	Eastern	Subjective evaluations	Journal	AA&AO	1.27
Zhou et al. (2014)	285	Primary school	Eastern	Subjective evaluations	Journal	AA&AO	1.13
Zhang et al. (2019)	3,012	Primary school	Eastern	Blank	Journal	SE&SS	1.36
Zhang et al. (2019)	3,012	Primary school	Eastern	Blank	Journal	SE&SS	1.36
Luo and Chen (2013)	252	Middle school	Eastern	Blank	Journal	SE&AA	0.76
Luo and Chen (2013)	252	Middle school	Eastern	Blank	Journal	SE&AA	0.76
Lu (2024)	696	Middle school	Eastern	Blank	Master dissertation	SS&AA	1.25
Lu (2024)	696	Middle school	Eastern	Blank	Master dissertation	SS&AA	1.25
Lu (2024)	696	Middle school	Eastern	Blank	Master dissertation	SS&AA	1.25
Mo (2023)	522	Middle school	Eastern	Blank	Master dissertation	SS&AA	0.89
Zychinski and Polo (2011)	133	Primary school	Western	Subjective evaluations	Journal	SE&AO	1.38
Karakus (2016)	126	Primary school	Western	Subjective evaluations	Doctor dissertation	SE&AO	1.00
Pajares and Valiante (2001)	497	Middle school	Western	Subjective evaluations	Journal	SE&AO	0.99
Gonida and Cortina (2014)	282	Middle school	Western	Subjective evaluations	Journal	SE&AO	1.17
Zhou et al. (2019)	238	Middle school	Western	Subjective evaluations	Journal	SE&AO	1.38
Barber (2010)	210	College	Western	Subjective evaluations	Doctor dissertation	SE&AO	blank
Los and Schweinle (2019)	315	College	Western	Objective measures	Journal	SE&AO	0.60
Azar et al. (2010)	280	College	Western	Subjective evaluations	Journal	SE&AO	0.14
Li et al. (2024)	483	Middle school	Eastern	Subjective evaluations	Journal	SE&AO	0.94
Li et al. (2024)	483	Middle school	Eastern	Subjective evaluations	Journal	SE&AO	0.94
Li et al. (2024)	483	Middle school	Eastern	Subjective evaluations	Journal	SE&AO	0.94
Kristensen et al. (2023)	750	Middle school	Western	Subjective evaluations	Journal	SE&SS&AO	0.96
Kristensen et al. (2023)	750	Middle school	Western	Subjective evaluations	Journal	SE&SS&AO	0.96
Kristensen et al. (2023)	750	Middle school	Western	Subjective evaluations	Journal	SE&SS&AO	0.96
Kristensen et al. (2023)	750	Middle school	Western	Subjective evaluations	Journal	SE&SS&AO	0.96
Kristensen et al. (2023)	750	Middle school	Western	Subjective evaluations	Journal	SE&SS&AO	0.96
Kristensen et al. (2023)	750	Middle school	Western	Subjective evaluations	Journal	SE&SS&AO	0.96
Luo et al. (2023)	1,158	College	Eastern	Objective measures	Journal	SE&AO	0.89
Fakhrou and Habib (2022)	43	College	Western	Subjective evaluations	Journal	SE&AO	1.69
Chun et al. (2025)	385	College	Eastern	Objective measures	Journal	SE&AO	0.89
Wang et al. (2024)	749	Middle school	Eastern	Subjective evaluations	Journal	SE&AO	1.02
Putwain et al. (2013)	206	College	Western	Subjective evaluations	Journal	SE&AO	0.47
Putwain et al. (2013)	206	College	Western	Subjective evaluations	Journal	SE&AO	0.47
Çakir and Işmen Gazioğlu (2021)	334	Middle school	Western	Subjective evaluations	Journal	SE&AO	1.32
Çakir and Işmen Gazioğlu (2021)	202	Middle school	Western	objective measures	Journal	TSS&AO	1.56
Çakir and Işmen Gazioğlu (2021)	202	Middle school	Western	objective measures	Journal	PeSS&AO	1.56
Dupont et al. (2015)	226	College	Western	Subjective evaluations	Journal	TSS&AO	0.45
Dupont et al. (2015)	226	College	Western	Subjective evaluations	Journal	PeSS&AO	0.45
Dupont et al. (2015)	226	College	Western	Subjective evaluations	Journal	PSS&AO	0.45
Chen (2005)	270	Middle school	Eastern	Subjective evaluations	Journal	PSS&AO	1.29
Chen (2005)	270	Middle school	Eastern	Subjective evaluations	Journal	TSS&AO	1.29
Chen (2005)	270	Middle school	Eastern	Subjective evaluations	Journal	PeSS&AO	1.29
Huang and Wang (2023)	651	College	Eastern	objective measures	Journal	TSS&AO	0.43
Zhang and Qian (2024)	265	Middle school	Eastern	Subjective evaluations	Journal	SS&AO	1.07
Lei et al. (2022)	845	Middle school	Eastern	Subjective evaluations	Journal	SE&SS&AO	0.78
Lei et al. (2022)	845	Middle school	Eastern	Subjective evaluations	Journal	SE&SS&AO	0.78
Li et al. (2018)	262	College	Eastern	Subjective evaluations	Journal	SS&AO	0.70
Qian et al. (2024)	2,654	Middle school	Eastern	Subjective evaluations	Journal	PSS&AO	1.07
Qian et al. (2024)	2,654	Middle school	Eastern	Subjective evaluations	Journal	TSS&AO	1.07
Qian et al. (2024)	2,654	Middle school	Eastern	Subjective evaluations	Journal	PeSS&AO	1.07
Möcklinghoff et al. (2023)	746	College	Western	Subjective evaluations	Journal	AA&AO	0.76
Zhou et al. (2020)	1,667	College	Eastern	Subjective evaluations	Journal	SE&AA&AO	1.22
Zhou et al. (2020)	1,667	College	Eastern	Subjective evaluations	Journal	SE&AA&AO	1.22
Zhang and Qian (2024)	2056	College	Eastern	Subjective evaluations	Journal	AA&AO	0.39
Rahafar et al. (2016)	158	Middle school	Western	Subjective evaluations	Journal	AA&AO	0.74
Legkauskas et al. (2019)	389	Primary school	Western	objective measures	Journal	AA&AO	0.81
Legkauskas et al. (2019)	389	Primary school	Western	objective measures	Journal	AA&AO	0.81
Kurdi (2017)	350	Primary school	Western	objective measures	Journal	AA&AO	1.08
Kurdi (2017)	350	Primary school	Western	objective measures	Journal	AA&AO	1.08
Chen et al. (2025)	1,119	College	Eastern	Blank	Journal	SE&SS	0.97
Chen et al. (2025)	1,119	College	Eastern	Blank	Journal	SE&SS	0.97
Thomas et al. (2017)	343	College	Western	Subjective evaluations	Journal	AA&AO	0.24
Balogun et al. (2017)	393	College	Western	Subjective evaluations	Journal	AA&AO	0.24
Palos et al. (2019)	254	College	Western	Subjective evaluations	Journal	SE&AO	3.79
Lüftenegger et al. (2015)	210	Middle school	Western	Subjective evaluations	Journal	SE&AO	0.98
Hunsu et al. (2023)	171	College	Western	Subjective evaluations	Journal	SE&AO	blank
Ollfors and Andersson (2022)	915	Middle school	Eastern	Subjective evaluations	Journal	SE&AO	0.87
Bubić et al. (2015)	361	Middle school	Eastern	Subjective evaluations	Journal	SE&AO	0.53
Shi et al. (2022)	262	Middle school	Eastern	Subjective evaluations	Journal	SS&AO	1.15
Shi et al. (2022)	262	Middle school	Eastern	Subjective evaluations	Journal	TSS&AO	1.15
Shi et al. (2022)	262	Middle school	Eastern	Subjective evaluations	Journal	PSS&AO	1.15
Shi et al. (2022)	262	Middle school	Eastern	Subjective evaluations	Journal	PeSS&AO	1.15
Li et al. (2023)	402	Middle school	Eastern	Subjective evaluations	Journal	TSS&AO	0.65
Li et al. (2023)	402	Middle school	Eastern	Subjective evaluations	Journal	TSS&AO	0.65
Li et al. (2023)	402	Middle school	Eastern	Subjective evaluations	Journal	TSS&AO	0.65
Fang et al. (2020)	2,328	Middle school	Eastern	Subjective evaluations	Journal	PSS&AO	1.14
Fang et al. (2020)	2,328	Middle school	Eastern	Subjective evaluations	Journal	PeSS&AO	1.14
Fang et al. (2020)	2,328	Middle school	Eastern	Subjective evaluations	Journal	TSS&AO	1.14

SE = Self-Efficacy; SS = Social support; PSS = Parental Social Support; TSS = Teacher Social Support; PeSS = Peer Social Support; AA = Academic anxiety; AO = Academic Outcomes; N = sample size.

### 3.4 Statistical methods

In this study, the correlation coefficient *r* was chosen as the effect size for the meta-analysis because it is a unitless measure and is widely used in social science research. We conducted effect size transformation and aggregation, heterogeneity testing, publication bias assessment, and moderator analyses on all reported *r* values. According to the method recommended by Hedges and Olkin (1985), the coefficients r extracted from each study were subjected to Fisher’s *Z* transformation to calculate the weighted average effect value for each group of relationships. The conversion formula between *r* and Fisher’s *Z* is as follows.


$${\text{Fisher}}^{’}s\;Z=0.5*\ln(\frac{1+r}{1-r}),V_{z}=\frac{1}{N-3},{\mathrm{SE}}_{z}=\sqrt{V_{z}},W=N-3$$

The heterogeneity of the aggregated effect sizes was assessed using Cochran’s Q statistic and the I^2^ index (with *I*^2^ < 25% indicating low heterogeneity, 25–50% moderate, 50–75% high, and ≥75% very high heterogeneity). When differences in sample characteristics or contextual factors were present across studies, a random-effects model was employed to obtain more robust estimates (Cooper et al., 2009). In addition to point estimates of effect sizes, 95% confidence intervals (CIs) for estimated correlation coefficients should also be reported. Three methods were used to assess publication bias. Firstly, the fail-safe N was calculated to estimate the number of unpublished non-significant studies required to render the overall effect non-significant (Orwin, 1983). A risk of publication bias is considered low if *N* > 5 *k* + 10 (where *k* is the number of studies) or exceeds 100. Secondly, funnel plots were generated to visually inspect symmetry, a largely symmetrical plot indicates robustness and the absence of publication bias. Thirdly, the Egger’s regression test was conducted to examine whether the results were affected by publication bias. In this method, if the linear regression coefficient is not statistically significant, it is considered that no publication bias exists (Egger et al., 1997).

Potential moderator effects were tested using a mixed-effects model, which applied random-effects models within each subgroup and a fixed-effects model to compare differences between subgroups.

To further examine the mediating role of SE and the overall pathways among the variables, we used SEM. The model was estimated in Amos, which makes it possible to analyze direct and indirect relationships among SE, SS, AA, and AO within a single framework.

## 4 Results

A total of 944 documents were retrieved, of which 59 (95 effect sizes) were ultimately included in the meta-analysis. The total sample size was 49,072, concentrated between the ages of 7 and 30, all of whom were students from primary school to university. The included studies that were mainly conducted in China and the United States.

### 4.1 Homogeneity tests

As shown in Table 2, the test indicated that the results included in the study were heterogeneous (*p* < 0.001), further proving the rationality of using a random effects model. The *I*^2^ value for all groups reached 75%, indicating significant heterogeneity between effect sizes and the possible presence of moderating variables that could potentially moderate the effect size. Therefore, a moderating effect test was necessary.

**Table 2:** Effect sizes for self-efficacy, social support, academic anxiety, and academic outcomes.

Variable relation	*k*	*r*	95% CI		*Z*	*p*	Qw	df(Q)	*I* ^2^
			Lower limit	Upper limit					
SE&AO	33	0.354	0.288	0.418	9.777	0.000	708.799***	32	95.479
SS&AO	34	0.245	0.200	0.289	10.441	0.000	611.549***	33	94.604
AA&AO	13	−0.179	−0.248	−0.109	−4.937	0.000	153.104***	12	92.162
SE&SS	7	0.494	0.429	0.555	12.799	0.000	70.316***	6	91.467
SE&AA	5	−0.321	−0.461	−0.166	−3.950	0.000	120.197***	4	96.672
SS&AA	4	−0.20	−0.273	−0.123	−5.068	0.000	12.340**	3	75.690

SE = Self-Efficacy; SS = Social support; AA = Academic anxiety; AO = Academic Outcomes; *k* = number of studies; Qw = within-group heterogeneity; df(Q) = the degree of freedom; *I*^2^ = the proportion of heterogeneity in the total variation in effect size.

### 4.2 Publication bias test

Across all analyses, the funnel plots showed that most effect sizes were concentrated in the upper-middle region and symmetrically distributed on both sides of the overall effect size (see Supplementary materials). The Egger’s regression tests and fail-safe N values for all analyses (Table 3) exceeded the critical threshold of 5 *k* + 10, indicating that the results were generally robust. Although the Egger’s regression test for the relationship between SE and AA was significant (*p* = 0.01), suggesting a potential publication bias, the large fail-safe N and the symmetrical pattern of the funnel plot indicate that the overall findings are relatively stable and unlikely to be overturned by unpublished null studies. Given the limited number of included studies (*k* = 5) and the low statistical power of Egger’s test in small-sample meta-analyses, publication bias cannot be entirely ruled out. Nevertheless, the main conclusions of this meta-analysis remain robust and reliable.

**Table 3:** Egger’s test results and fail-safe N.

Variable relation	Intercept	95% CI		*p*	Fail-safe N
		Lower limit	Upper limit		
SE&AC	−1.98	−7.20	3.24	0.45	7,561
SS&AC	−0.21	−3.32	2.91	0.89	5,048
AA&AC	1.56	−4.09	7.21	0.56	1,076
SE&SS	0.79	−10.05	11.63	0.86	3,558
SE&AA	−12.24	−17.54	−6.93	0.01	348
SS&AA	−16.5	−89.67	56.61	0.43	105

SE = Self-Efficacy; SS = Social support; AA = Academic anxiety; AO = Academic Outcomes; p < 0.05 indicates statistical significance; 95% CI = 95% confidence interval.

### 4.3 Main effects test

From Table 2, we can see that the mean weighted correlation coefficients between SE, SS, and AA with AO are 0.354, 0.245, and −0.179, respectively. This indicates that SE, SS, and AA exert a certain degree of influence on AO, supporting H1. We can also observe that the average weighted correlation coefficient between SE and SS reached 0.494, while the average weighted correlation coefficient between SE and AA reached −0.321. SS and AA exhibited a correlation coefficient of −0.2. Z-values and two-tailed *p*-values indicate the statistical significance of point estimates. According to Cohen’s criteria, |*r*| ≤ 0.10 indicates a small effect size, |*r*| = 0.30 indicates a moderate effect size, and |*r*| ≥ 0.50 indicates a large effect size. The study found that AA exhibits a moderate to low negative correlation with AO and SS. There is a moderate to high positive correlation between AA and SE. SS exhibits a moderate to low positive correlation with AO, showing a moderate to low positive correlation. Additionally, the average weighted correlation coefficients between SE and AO, as well as SS, are 0.354 and 0.494, respectively. These coefficients are statistically significant, indicating a moderate to high positive correlation. There is a moderate to low positive correlation between SS and AO.

### 4.4 Moderating effects test

To examine the moderating effects on the relationships among the four variables, studies were categorized into subgroups by grade level (primary, secondary, and higher education), cultural background (Eastern vs. Western), the types of AO measurement (objective measures vs. subjective evaluations), and the types of SS into three subgroups (Parent support, Teacher Support, and Peer Support). Studies that could not be classified into any subgroup were excluded from the analyses (Tables 4–7).

**Table 4:** Effects of different cultural backgrounds on the four variables.

Group	ES	*g*	95%CI	*Q*-value	df(Q)	Meta-regression
SE → AO						
Eastern	15	0.377***	[0.269,0.476]	0.533	1	ns
Western	18	0.331***	[0.261,0.397]			
SS → AO						
Eastern	26	0.261***	[0.209,0.311]	4.910	1	*
Western	8	0.182***	[0.134,0.229]			
AA→AO						
Eastern	5	−0.229***	[−0.316,-0.139]	1.406	1	ns
Western	8	−0.145**	[−0.251,-0.0035]			
SE → SS						
Eastern	4	0.501***	[0.413,0.580]	0.045	1	ns
Western	3	0.485***	[0.351,0.599]			

SE = Self-Efficacy; SS = Social support; AA = Academic anxiety; AO = Academic Outcomes; ***p < 0.001, **p < 0.01, *p < 0.05. ES = number of effect sizes; g = pooled effect size; 95% CI = 95% confidence interval; Q = the heterogeneity value; df(Q) = the degree of freedom; ns = non-significant.

**Table 5:** Effects of different grade level on the four variables.

Group	ES	*g*	95%CI	*Q*-value	df(Q)	Meta-regression
SE → AO						
Primary School	3	0.290***	[0.229,0.348]	3.962	2	ns
Secondary School	17	0.315***	[0.244,0.382]			
College	13	0.420***	[0.306,0.523]			
SS → AO						
Primary School	1	0.250***	[0.228,0.272]	8.902	2	*
Secondary School	28	0.258***	[0.204,0.311]			
College	5	0.173***	[0.125,0.211]			
AA→AO						
Primary School	5	−0.144**	[−0.239,-0.046]	1.097	2	ns
Secondary School	3	−0.224***	[−0.337,-0.104]			
College	5	−0.190**	[−0.324,-0.048]			
SE → SS						
Primary School	1	0.460***	[0.431,0.488]	36.989	2	***
Secondary School	5	0.425***	[0.396,0.547]			
College	1	0.679***	[0.572,0.646]			

SE = Self-Efficacy; SS = Social support; AA = Academic anxiety; AO = Academic Outcomes; ***p < 0.001, **p < 0.01, *p < 0.05; ES = number of effect sizes; g = pooled effect size; 95% CI = 95% confidence interval; Q = the heterogeneity value; df(Q) = the degree of freedom; ns = non-significant.

**Table 6:** Effects of different types of academic outcomes measurement on the four variables.

Group	ES	*g*	95%CI	Q-value	df(Q)	Meta-regression
SE → AO						
Objective measures	6	0.526***	[0.413,0.624]	10.985	1	**
Subjective evaluations	27	0.308***	[0.253,0.360]			
SS → AO						
Objective measures	6	0.305***	[0.224,0.382]	2.295	1	ns
Subjective evaluations	28	0.232***	[0.182,0.281]			
AA→AO						
Objective measures	4	−0.135**	[−0.253,-0.013]	0.707	1	ns
Subjective evaluations	9	−0.198***	[−0.281,-0.112]			

SE = Self-Efficacy; SS = Social support; AA = Academic anxiety; AO = Academic Outcomes; ***p < 0.001, **p < 0.01, *p < 0.05; ES = number of effect sizes; g = pooled effect size; 95% CI = 95% confidence interval; Q = the heterogeneity value; df(Q) = the degree of freedom; ns = non-significant.

**Table 7:** Effects of different types of social support on academic outcomes.

Group	ES	*g*	95%CI	*Q*-value	df(Q)	Meta-regression
SS → AO						
Parent support	7	0.227***	[0.128,0.321]	0.525	2	ns
Teacher support	12	0.228***	[0.147,0.307]			
Peer support	9	0.270***	[0.171,0.364]			

SS = Social support; AO = Academic Outcomes; ***p < 0.001, **p < 0.01, *p < 0.05; ES = number of effect sizes; g = pooled effect size; 95% CI = 95% confidence interval; Q = the heterogeneity value; df(Q) = the degree of freedom; ns = non-significant.

A significant positive association was consistently observed among SE, SS, and AO (*p* < 0.01) across all analyzed grade levels, cultural backgrounds, and types of AO measurement. Conversely, AA demonstrated a significant negative association with AO (*p* < 0.01). Cultural background significantly moderated the association between SS and AO (*p* < 0.05), with students in Eastern cultural contexts exhibiting a slightly stronger relationship than those in Western contexts. While the association between SE and AO showed a modest upward trend across grade levels, subgroup differences were not statistically significant (*p* > 0.05). Grade level significantly moderated the association between SS and AO (*p* < 0.05). The strongest effect was found among middle school students (*r* = 0.258), followed by primary school students (*r* = 0.250), and was weakest among college students (*r* = 0.173). Grade level also significantly moderated the SS and SE association (*p* < 0.05), with the strongest effect observed among college students (*r* = 0.679), followed by primary (*r* = 0.460) and middle school students (*r* = 0.425). The type of AO measurement was a significant moderator (*p* < 0.01) for SE, showing a stronger positive association with objective measures (*r* = 0.526) than with subjective evaluations (*r* = 0.308). Although SS and AA maintained their respective positive and negative associations across both measurement subgroups, these differences were not significant. Finally, all three types of SS were positively associated with AO with comparable effect sizes, indicating that the type of SS does not meaningfully moderate this relationship.

### 4.5 Mediating role of social support and academic anxiety

**Figure 2:**
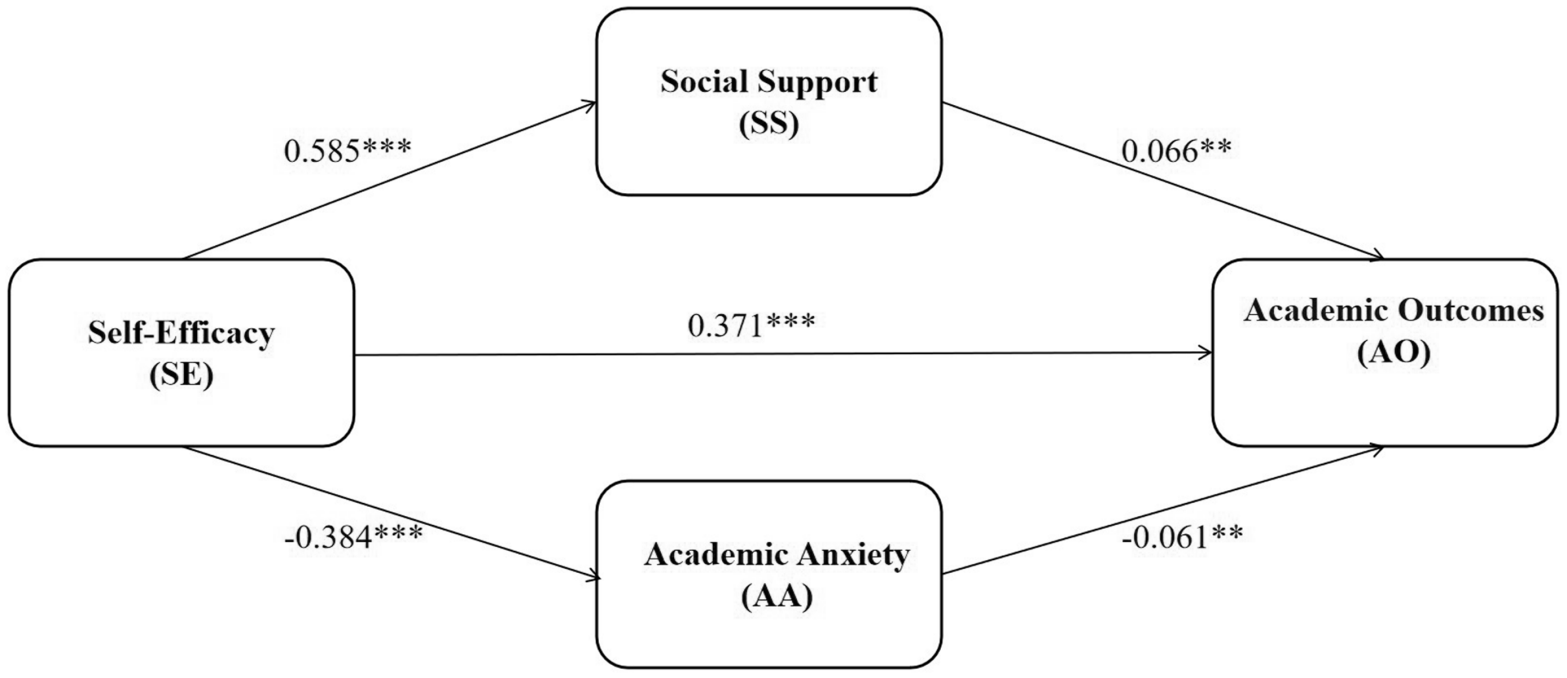
Mediating role of social support and academic anxiety.

Structural equation modeling was used to examine how the variables in this study are related. In the model, AO were the dependent variable, SE was the independent variable, and SS and AA were included as mediators (see Figure 2). SEM was chosen because it makes it possible to analyze several relationships at the same time and to estimate the direct and indirect effects among variables while taking measurement error into account. This allows for a clearer understanding of how SE may influence AO through perceived SS and AA. The mediating hypotheses were tested based on this model. The overall model fit was evaluated using commonly recommended indices (see Table 8). The results showed that the model fit the data very well: χ^2^/df = 0.739, RMR = 0.003, GFI = 1.000, AGFI = 0.999, NFI = 1.000, TLI = 0.999, CFI = 1.000, and RMSEA = 0.000. All values met the accepted criteria, indicating that the model provided an excellent representation of the observed data.

**Table 8:** Validation factor model fit.

Indicators	χ^2^/df	RMR	GFI	AGFI	NFI	TLI	CFI	RMSEA
Results	0.739	0.003	1.000	0.999	1.000	1.000	1.000	0.000
Standards	<3	<0.1	>0.9	>0.9	>0.9	>0.9	>0.9	<0.05
Situation	Fit	Fit	Fit	Fit	Fit	Fit	Fit	Fit

Figure 2 demonstrates that SE exhibits a significant negative correlation with AC (*γ* = 0.371, *p* < 0.001), further confirming Hypothesis H1. Additionally, SE exhibits a significant positive correlation with SS (γ = 0.585, *p* < 0.001) and a significant negative correlation with AA (γ = −0.384, *p* < 0.001). SS has a significant positive effect on AC (γ = 0.066, *p* < 0.01), while AA has a significant negative effect on AC (γ = −0.061, p < 0.01).

To examine whether SS and AA mediate the effect of SE on AO, we utilized the Bootstrap procedure as implemented in Amos. This robust statistical method estimates indirect effects by repeatedly resampling the data and calculating 95% confidence intervals. A mediating effect is considered statistically significant when its corresponding 95% confidence interval does not include zero. The resulting Bootstrap estimates for all mediating pathways are systematically presented in Table 9.

**Table 9:** Mediating effect test of social support and academic anxiety.

Path	Effect value	SE	95% CI		*p*	Mediation
			Lower limit	Upper limit		
Total effect	0.432	0.016	0.402	0.463	<0.001	100%
Direct effect	0.371	0.024	0.323	0.419	<0.001	85.88%
Indirect effect	0.062	0.016	0.030	0.091	<0.001	14.35%
SE → SS → AO	0.038	0.013	0.011	0.064	0.005	8.80%
SE → AA→AO	0.023	0.008	0.008	0.039	0.002	5.32%

SE = Self-Efficacy; SS = Social support; AA = Academic anxiety; AO = Academic Outcomes; ***p < 0.001, **p < 0.01, *p < 0.05; 95% CI = 95% confidence interval.

According to Table 9, the analysis revealed that both SS and AA play a significant mediating role in the pathway from SE to AO. The total indirect effect was calculated to be 0.062, which collectively constitutes 14.35% of the overall effect. These findings provide strong empirical support for Hypothesis H3.

Specifically, SS demonstrated a significant positive indirect effect in the link between SE and AO. Its 95% confidence interval did not encompass zero, and the standardized indirect effect was 0.038, accounting for 8.8% of the total effect. Correspondingly, AA also exhibited a significant mediating effect, with its 95% confidence interval similarly excluding zero. The standardized indirect effect for AA was 0.023, contributing 5.32% of the total effect.

## 5 Discussion

### 5.1 Relationship between self-efficacy and academic outcomes

Our findings indicate that SE was significantly and positively associated with AO (*r* = 0.354), supporting its role as a key factor in enhancing students’ AO. Consistent with SE theory, individuals with higher SE tend to engage more persistently with academic tasks and are more capable of overcoming challenges through sustained effort (Bandura, 1977), which in turn contributed to improved AO. The magnitude of the effect observed in this study is highly consistent with prior meta-analyses and empirical research. Carpenter (2007) research encompassed a large higher education sample, reported a correlation coefficient of 0.345 between SE and AO, virtually identical to the present findings. Yi et al. (2017) similarly identified a correlation coefficient of 0.33, while Ji and Zhao (2021) observed a slightly higher coefficient (*r* = 0.381). This convergence across diverse samples provides robust evidence for the stable predictive role of SE in AO. At the same time, individual primary studies show variability. Li et al. (2024) found a comparatively strong association between general SE and secondary school students’ AO (*r* = 0.47), while Wang et al. (2024) found only a weak correlation between subject-specific SE in geography and geography grades (*r* = 0.09). From the perspective of social cognitive theory, such discrepancies can be understood through the principle of “domain specificity,” which posits that SE exerts the strongest predictive effect when the efficacy domain aligns directly with the outcome domain (Pajares, 1996). Li et al. (2024) general SE corresponded with the broadly measured AO, whereas Wang et al. (2024)‘s geography-specific efficacy may have been confounded by unmeasured domain-specific factors like prior geographical knowledge or subject interest. By integrating evidence across domains and samples in the present meta-analysis, we confirm that although domain specificity contributes to variations in effect size, the positive association predicted by social cognitive theory remains consistent and theoretically robust.

### 5.2 Moderating factors

The present study confirms that SE and SS positively impact the AO of adolescents, while AA exerts a negative influence, effects that generally hold irrespective of grade level and broad cultural background (Kenyatta University and Masisi, 2024; Rahafar et al., 2016; Yi et al., 2017). A notable common trend emerged, the correlation effect sizes for SE, SS, and AA with AO tend to be larger in Eastern cultural contexts than in Western ones. This pattern is potentially attributed to the increased emphasis on collectivism in Eastern culture, where collective interests often supersede individual interests, and individual behavior is profoundly influenced by group norms and social expectations (Markus and Kitayama, 1991). Consequently, students’ academic SE frequently intertwines with the high expectations of their families and communities, turning high SE into a means of fulfilling the collective pursuit of academic excellence and thus strengthening its predictive power. Specifically, cultural differences significantly moderated the effect of SS on AO, with a stronger association observed in Eastern samples. This suggests that SS in these contexts not only offers emotional comfort but also conveys crucial social norms and behavioral guidance, which is more readily translated into concrete academic engagement. Conversely, Western cultures, prioritizing individual autonomy and intrinsic motivation, may rely more on self-driven effort, which could diminish the reinforcing role of external relational support. Interestingly, the relationship between SE and AO did not exhibit a significant cultural moderation effect, which might suggest a universal function for SE as an internal belief that enhances learning motivation, persistence, and strategic behaviors across cultures. However, this non-significant finding may also reflect methodological limitations, such as heterogeneity arising from variations in sample composition, measurement instruments for SE and AO, academic subjects, and educational systems. Such factors could potentially obscure subtle cultural differences. Therefore, future research is warranted to employ individual-level measures of cultural orientation rather than national-level indicators to more precisely capture the influence of culture on these underlying psychological mechanisms.

SS and AO show different degrees of correlation in different grades. The relationship between the two is the closest among middle school students, followed by primary school students, and the lowest among college students. This is similar to previous studies. Levitt studied students from grades 1–2, 4–5, and 8–9. The study found that with the increase of age, the impact of SS on AO gradually increases (Levitt, 1992). In addition, Wentzel’s study also pointed out that there are differences in the relationship between SS and academic motivation among students of different grades. For example, peer support has less impact on AO in primary school, but more significant in middle school (Wentzel, 1998).

SE and SS showed significant differences in different grades. The relationship between the two was the closest in college students and the lowest in middle school students. This is different from Orejudo et al. (2021) viewpoint, which may be because there are fewer literatures conducting subgroup analysis in this study and Santos Orejudo focused on music students. According to Erikson (1950) psychological development theory, students in different grades are at different stages of development tasks. College students are facing the task of intimacy versus loneliness. SS comes more from peer groups, mentors and career-related networks, and the support content involves higher order needs such as academic autonomy and career planning. At this time, the core of SE is the confidence to achieve goals independently. SS directly strengthens SE by providing emotional resonance and information resources, so the regulatory effect is the strongest. Primary school students are in the stage of diligence versus inferiority, and SS mainly comes from parents and teachers, and the support form is mostly direct learning guidance and emotional encouragement. Immediate SS such as parental tutoring homework and teacher praise has a significant impact on them. For middle school students who face identity versus role confusion, SS shifts significantly toward peer groups. However, their drive for independence often conflicts with external support, thereby reducing its acceptance. As academic complexity increases, SE formation becomes reliant on autonomous exploration, weakening the moderating effect of SS due to the tension between autonomy needs and external intervention. The non-significant findings for some moderator effects may be related to uneven sample distributions and limitations in study design. Most included studies were cross sectional, making it difficult to capture developmental changes, and inconsistencies in grade level classifications across studies may have introduced additional statistical error. Future research could use longitudinal designs or growth curve modeling to examine whether these stage related differences follow nonlinear trajectories. For example, whether the effect of SS peaks during adolescence and then declines, and whether the translation of SE into proactive help-seeking strengthens with age.

The subgroup analysis based on the types of AO measurement showed that SE was positively associated with AO in both objective measures and subjective evaluation, with a significantly stronger effect in the objective measures group. This suggests that the types of AO measurement moderates the relationship between SE and AO. Consistent with previous research, SE positively predicts AO (Cai and Yang, 2019; Li et al., 2024). However, unlike prior studies, the present study refined the types of AO measurement by distinguishing between objective measures and subjective evaluations. We found that the predictive power of SE was stronger under objective measures. This result indicates that the influence of SE is more likely to manifest in tasks that are quantifiable and have clear evaluation criteria, as individuals’ beliefs in their abilities can more directly translate into learning behaviors and observable performance. In contrast, in subjective evaluation contexts, factors such as rater expectations, social comparison, and emotional biases may weaken this direct link, thereby reducing the effect. In comparison, the paths from SS to AO and from AA to AO did not differ significantly between objective and subjective grade groups. This may be because the effects of SS and AA on AO are often indirect, primarily operating through internal processes such as emotion regulation and cognitive processing (Ahmed et al., 2010). These psychological effects are relatively consistent and do not change drastically based on the measure type. Regardless of whether the types of AO measurement are objective measures or subjective evaluation, students who feel supported tend to feel more secure and less anxious, but the manifestation of these states is more likely to accumulate over longer-term adaptation rather than being tied to a single, specific type of evaluation. The non-significant moderation for SS and AA does not mean they are unimportant. It indicates their effects are indirect, steady, and less sensitive to the specific format of the performance measure. Future research could utilize both objective and subjective indicators within longitudinal and cross cultural designs to closely track how these psychological factors operate across different time frames and assessment systems.

Although the subgroup analysis indicated only marginal differences in effect sizes across parental, teacher, and peer support, these variations ultimately did not reach statistical significance. One plausible theoretical explanation is that the functional core of SS, encompassing emotional reassurance, motivational encouragement, and the provision of practical or informational resources tends to exert a broadly similar influence on students’ AO, largely irrespective of the source. This shared fundamental mechanism may effectively overshadow any subtle variations associated with the specific social context. Additionally, methodological factors likely contribute to the lack of significant differentiation. The measurement of SS in primary studies often exhibits considerable variation in scale design, content, and granularity. Such inconsistencies may dilute subgroup distinctions and restrict the statistical power necessary to detect genuine between group differences. Another compounding factor is the substantial overlap among support types, as students reporting high support from one source frequently experience high support from others, making it challenging to statistically isolate the unique contribution of each subtype. Therefore, future research should employ more fine grained and source sensitive measures of SS to better differentiate its subtypes. Implementing longitudinal designs could further clarify whether the relative influence of parental, teacher, and peer support shifts across developmental stages. Moreover, incorporating cross cultural comparisons is crucial to reveal how contextual norms shape the salience of different support types. Finally, studies leveraging multi-informant reports or behavioral indicators, rather than solely relying on self-reported perceptions, may enhance the precision needed to detect true subgroup differences. These research directions will help clarify whether the non-significant differences observed here reflect a genuine theoretical convergence or are primarily due to methodological limitations in the existing literature.

### 5.3 Mediating role of social support and academic anxiety

This study revealed that SS and AA play a mediating role between SE and AO through mediation analysis. Specifically, students with higher SE tend to perceive more SS, which in turn improves AO. At the same time, students with higher SE generally experience lower levels of AA, remain calmer during examinations, and are more likely to perform successfully on exams.

SS significantly and positively predicted SS (*γ* = 0.585, *p* < 0.001), and SS further significantly and positively predicted AO (γ = 0.066, *p* < 0.01), forming a transmission chain of “SE → SS → AO,” with a standardized mediation effect value of 0.038, accounting for 8.8% of the total effect. This means that students with high SE are more likely to perceive support from their parents, teachers or peers, and this support is ultimately transformed into improved AO by providing emotional resonance and resource assistance. Different from previous studies that believed that SE plays a mediating role between SS and AO (Zhang et al., 2019), this study revealed through mediation analysis that SS plays a mediating role between SE and AO. Social Cognitive Theory helps explain the link between SE and perceived SS. According to Bandura (1986), SE influences how individuals select behaviors and interpret environmental feedback. Students with higher SE tend to be more confident in handling academic tasks and are more likely to interpret feedback from teachers or peers as encouragement and recognition. This positive appraisal enables them to perceive greater support from parents, peers, and teachers, and to actively engage in constructive social interactions. In contrast, students with lower SE may interpret the same feedback as criticism, resulting in lower perceived SS. Empirical studies indicate that students with higher SE are more likely to obtain and utilize SS resources effectively (Tang and Deng, 2014). Particularly in higher grades, mature SE acts as a core driver of academic behavior, prompting confident students to actively seek guidance from instructors, form study groups, and engage in academic discussions, thereby maximally leveraging SS for AO.

Social support significantly negatively predicted AA (γ = −0.384, *p* < 0.001), and AA significantly negatively predicted AO (γ = −0.061, *p* < 0.01), constituting the reverse conduction of “SE → AA→AO,” with a standardized mediation effect value of 0.023, accounting for 5.32% of the total effect. This shows that high SE indirectly promotes AO by suppressing the level of AA and reducing the interference of negative emotions on cognitive processes. This study found that mathematics SE can indirectly affect mathematics results through mathematics anxiety. Mathematics anxiety plays a partial mediating role in the relationship between mathematics SE and mathematics results. This result confirms the view of Gao and Chen (2017) hat mathematics anxiety can explain the impact of SE on mathematics results. According to Coping Theory, individuals with higher SE are more likely to adopt problem focused coping strategies when facing stress, rather than engaging in avoidance or passive responses (Folkman, 1997). They are more likely to seek information, consult teachers, or discuss learning challenges with peers. These behaviors help them access additional resources, reduce academic stress, and lower anxiety. Recent work has also shown similar patterns, where psychological factors influence students’ academic experiences through indirect pathways rather than direct effects (Öztekin et al., 2025). Empirical evidence shows that SE is positively associated with active coping behaviors, which effectively decrease anxiety levels. Reduced anxiety, in turn, improves attention and learning efficiency, ultimately leading to better AO.

In this study, the mediating effect of SS was stronger than that of AA. This suggests that helping students feel more supported may be a more effective way to boost their AO than just trying to lower their anxiety. Students who feel confident are more likely to actively seek help, join study groups, or participate in discussions. The support they receive during these interactions then further increases their confidence. This creates a positive cycle that not only helps reduce anxiety but also directly leads to better engagement and AO. Prior research also highlights the mediating role of SS in pathways linking stress with positive psychological outcomes, supporting the idea that support operates as a meaningful psychological resource (Yıldırım and Green, 2024). SS has further been associated with higher levels of resilience and psychological flexibility, reinforcing its role as a key protective factor in student well-being (Yıldırım and Aziz, 2023). SS offers important help and encouragement from outside, while lower anxiety allows students to focus better and stay calm. Through these two distinct paths, SE ultimately influences AO, with both SS and AA contributing to the significant overall positive effect.

It should be noted that, despite applying a random effects model to account for heterogeneity across studies, some of the moderation and mediation effects reported here may be subject to limited statistical power. This limitation is especially relevant in analyses involving smaller sample sizes or lower effect sizes, meaning that certain effects, particularly those of modest magnitude, may have been missed or estimated with reduced precision. This suggests that the current findings, while robust, could be more refined. Therefore, future research should utilize larger and more diverse samples and apply more sophisticated statistical approaches, such as multilevel modeling or refined subgroup analyses. Such efforts would significantly help to enhance the robustness and generalizability of these findings and provide a more precise understanding of the underlying mechanisms.

## 6 Implications

This meta-analysis study is based on ecosystem theory and incorporates social learning theory, using SS and AA as mediating variables to construct a relational model. It resolves controversies between individual studies and elucidates the underlying mechanisms of the relationship between SE and AO, which has been validated using SEM techniques. Additionally, the path analysis and the mediating effects of SS and AA were validated using SEM methods, marking a new endeavor in the application of this software in psychological research. The findings of this study elucidate the relationship between SE and AO, thereby providing a foundation and new perspective for exploring educational pathways to effectively enhance students’ AO levels and promote their physical and mental well-being.

The findings of this study offer several valuable practical suggestions for educational settings. Firstly, SS has been shown to be malleable and responsive to targeted interventions, as demonstrated by recent course-based programs that successfully enhanced students’ creative SS (Green et al., 2024b). Given the strong role of SE in predicting AO and its indirect influence via SS and AA, it is suggested that teachers and counselors should focus on structuring learning environments that help build stronger efficacy beliefs. This could involve offering appropriate challenges, providing timely and specific feedback, and creating opportunities for students to experience clear success and mastery. Secondly, the important mediating role of SS highlights the benefit of cultivating supportive networks. Schools might consider ways to strengthen students’ perceived and actual support, perhaps by encouraging peer study groups, setting up teacher support systems, and offering mentoring programs. Making counseling services easily accessible to address emotional and academic needs would also be beneficial. Thirdly, the link showing that SE helps to reduce AA suggests the value of integrating basic emotion regulation strategies into school routines. Simple guidance on study planning, training in managing emotions, and methods for reframing negative thoughts could assist students in handling pressure more effectively. Finally, the moderator effects remind us that intervention efforts may need to be tailored to developmental stages and cultural backgrounds. For instance, reinforcing SS networks might be most helpful for middle school students, while university students could benefit more from help in translating their SE into effective collaboration and resource use. Overall, these findings support the design of flexible, multilevel interventions that aim to enhance both student achievement and psychological well-being.

## 7 Limitations

Several limitations should be acknowledged when interpreting the findings of this meta-analytic structural equation model. First, most of the included studies relied on cross sectional designs, which restricts the ability to draw causal inferences regarding the relationships among SE, SS, AA, and AO. Future research would benefit from longitudinal or experimental designs that can better capture the temporal dynamics and causal pathways implied in the current model. Second, although the meta-analytic approach increases statistical power, potential sample bias remains a concern. The included studies varied in sample size, cultural background, and academic levels, and some subgroups (such as early childhood or vocational education) were underrepresented, which may limit the generalizability of the findings. Third, the model was constrained by the variables reported in the primary studies. Although other potential mediators, such as learning motivation, learning strategies, or emotion regulation, could provide additional insights into the mechanisms of AO, very few of the included studies measured these variables, making it unfeasible to include them in the current analysis. The absence of these unmeasured variables, along with other contextual factors such as family socioeconomic status, teacher student relationships, personality traits, or school climate, may have introduced omitted variable bias and reduced the explanatory precision of the model. Future directions can focused meta-analyses on motivation, resilience, or emotion regulation when more studies become available or systematic narrative reviews synthesizing existing evidence where quantitative aggregation is not yet feasible. Fourth, although publication bias was assessed, the reliance on published studies may still lead to an overestimation of effect sizes, especially for small sample research reporting significant associations. Future meta-analyses should integrate more gray literature and datasets to mitigate this issue. Finally, while the moderation analyses offer insights into cultural and grade level differences, finer grained moderators. Such as measurement instruments, academic domains, or assessment methods should be explored in future work to provide a more nuanced understanding of heterogeneity. Addressing these limitations through expanded datasets, longitudinal research, and inclusion of additional mediators and contextual variables will strengthen the robustness, accuracy, and practical value of subsequent studies.

## 8 Conclusion

This study used a meta-analytic structural equation model to clarify how SE, SS, AA, and AO are linked. We confirmed a moderate correlation between SE and AO (*r* = 0.354) and further showed that this relationship varies slightly by the type of AO measure, while the mediating roles of SS and AA remain largely stable across measurement types.

By incorporating dual mediators, the study integrated cognitive, social environmental, and emotional pathways, extending social cognitive theory toward a broader socio-ecological perspective. This aligned with recent findings showing that SS reliably mediates psychological and AO across diverse contexts (Aslan et al., 2025; Çiçek et al., 2024; Green et al., 2024a; Green et al., 2024b).

We also identified meaningful moderation effects: cultural background shaped the mechanisms of SS, and developmental stage showed complex trajectories rather than linear change. These results contributed to understanding how cultural and developmental factors jointly regulate the transformation of SE into AO.

Of course, this study has limitations, such as the limited number of studies simultaneously including four variables and the failure to cover all potential mediating variables. Future research should adopt longitudinal designs to test causal mechanisms more rigorously. Practically, the findings support developing differentiated, theory driven interventions that strengthen SE while accounting for cultural background and developmental stages. This provides a solid theoretical foundation and practical pathway for advancing the goal of high-quality education.

## Data Availability

The original contributions presented in the study are included in the article/supplementary material, further inquiries can be directed to the corresponding author/s.
