# Supplementary material for: Exploring the relationship between self-efficacy, social support, academic anxiety, and academic outcomes: a meta-analysis structural equation modeling approach
Source: Front Psychol. 2025 Dec 10;16:1714845. doi: 10.3389/fpsyg.2025.1714845 (PMC12727568; doi:10.3389/fpsyg.2025.1714845)
Supplement: Supplementary file 1 [file Image_1.pdf]

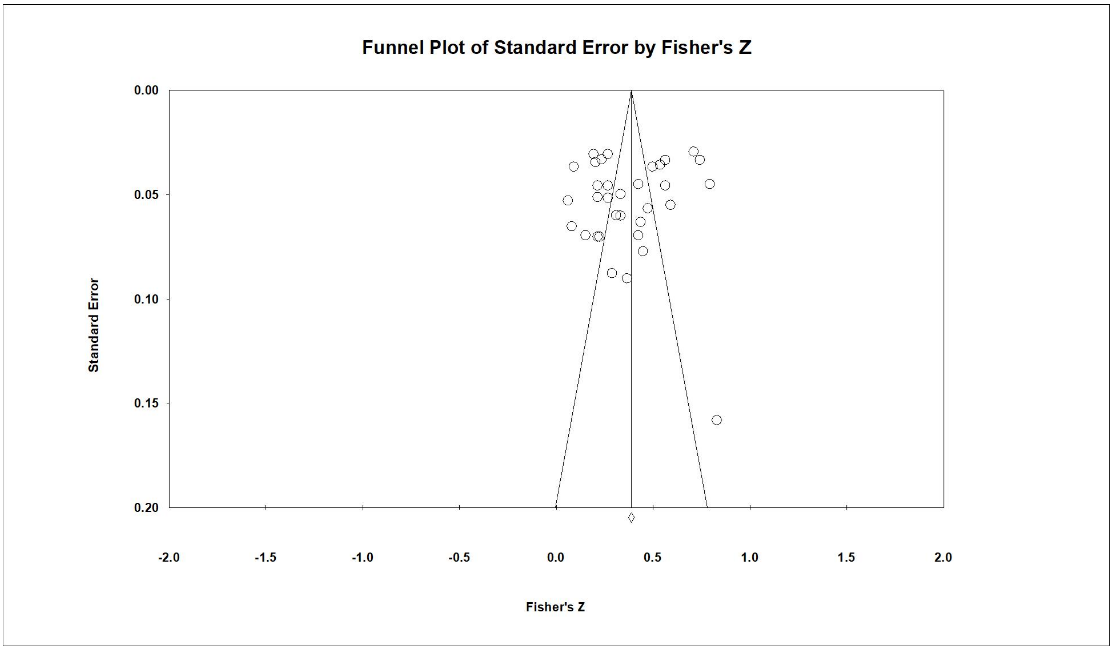

FIGURE 1 **Funnel plot of relation between self efficacy and academic outcomes**

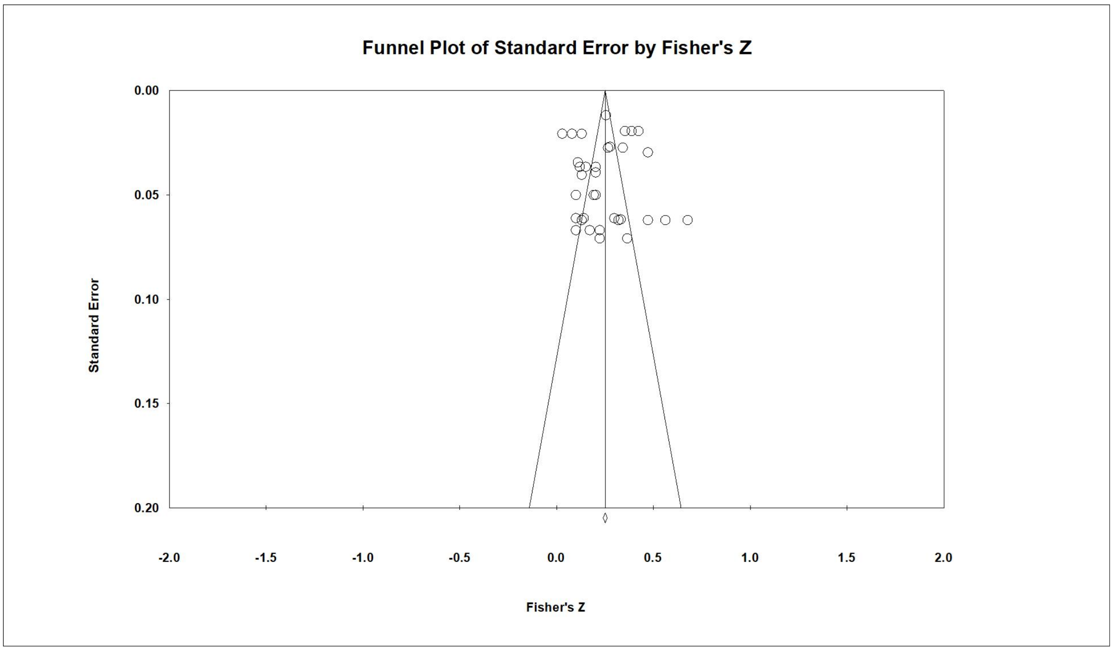

FIGURE 2 **Funnel plot of relation between social support and academic outcomes**

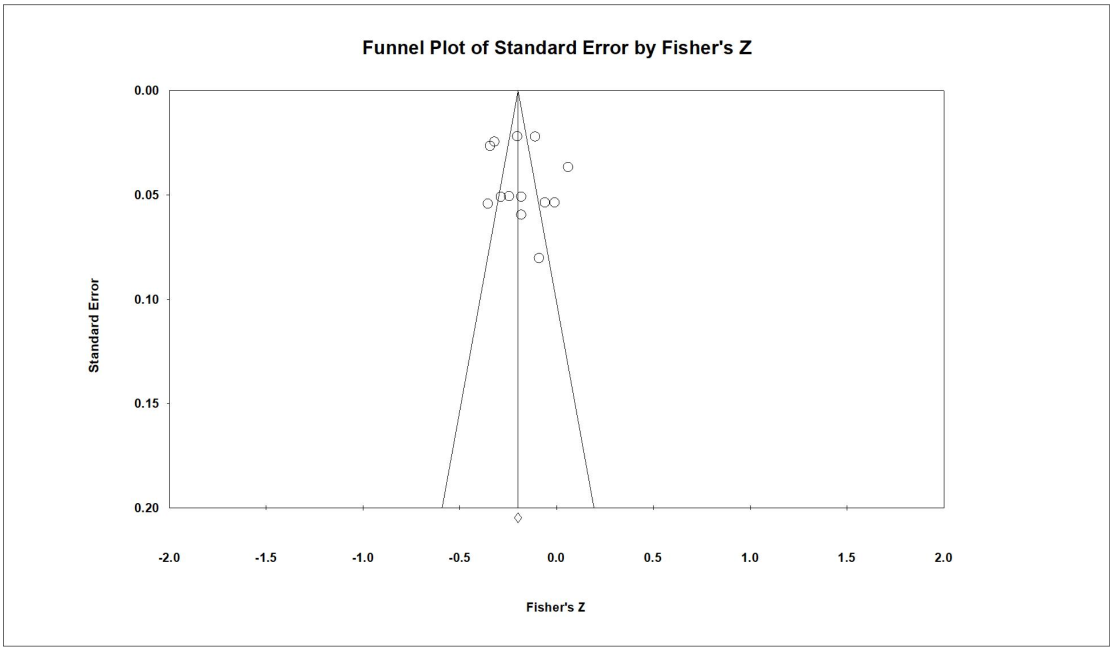

FIGURE 3 **Funnel plot of relation between academic anxiety and academic outcomes**

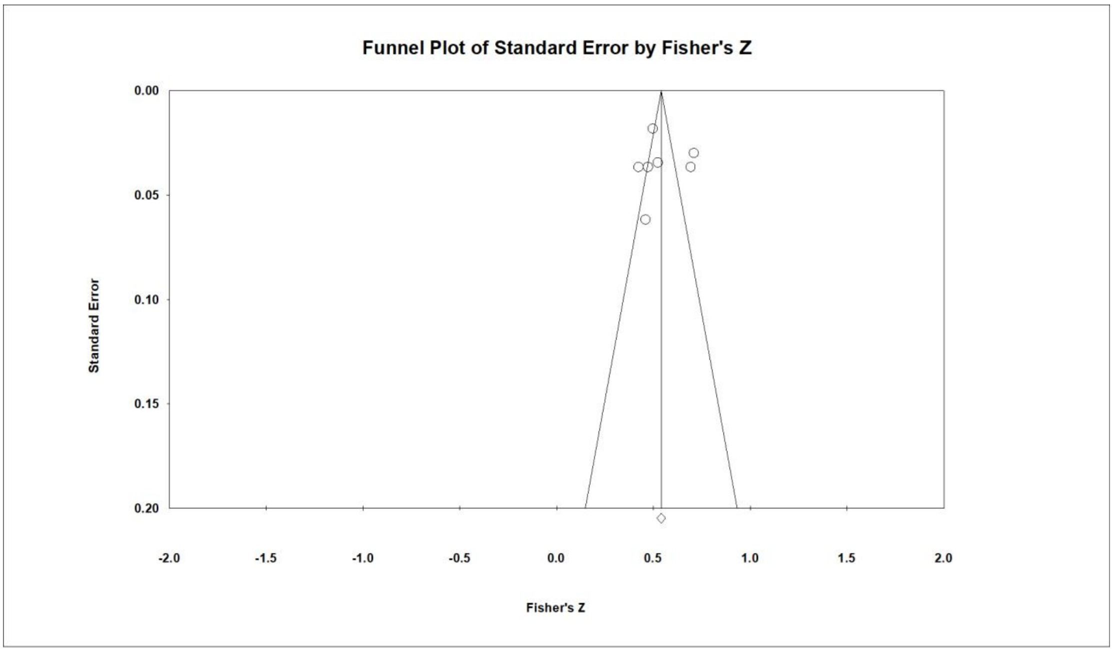

FIGURE 4 **Funnel plot of relation between self efficacy and social support**

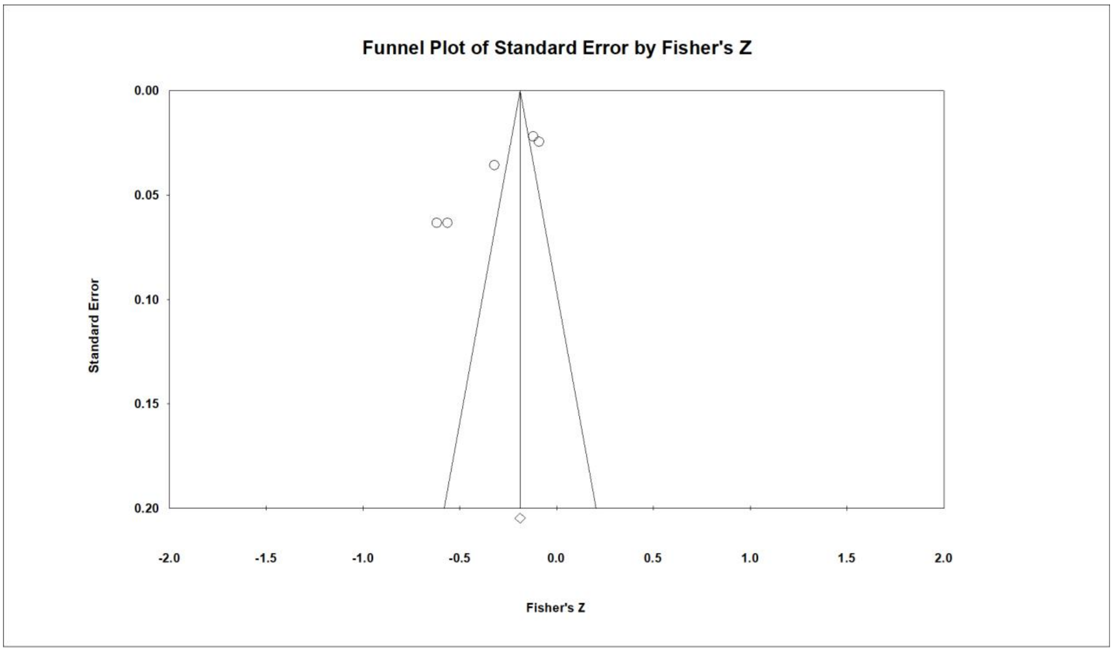

FIGURE 5 **Funnel plot of relation between self efficacy and academic anxiety**

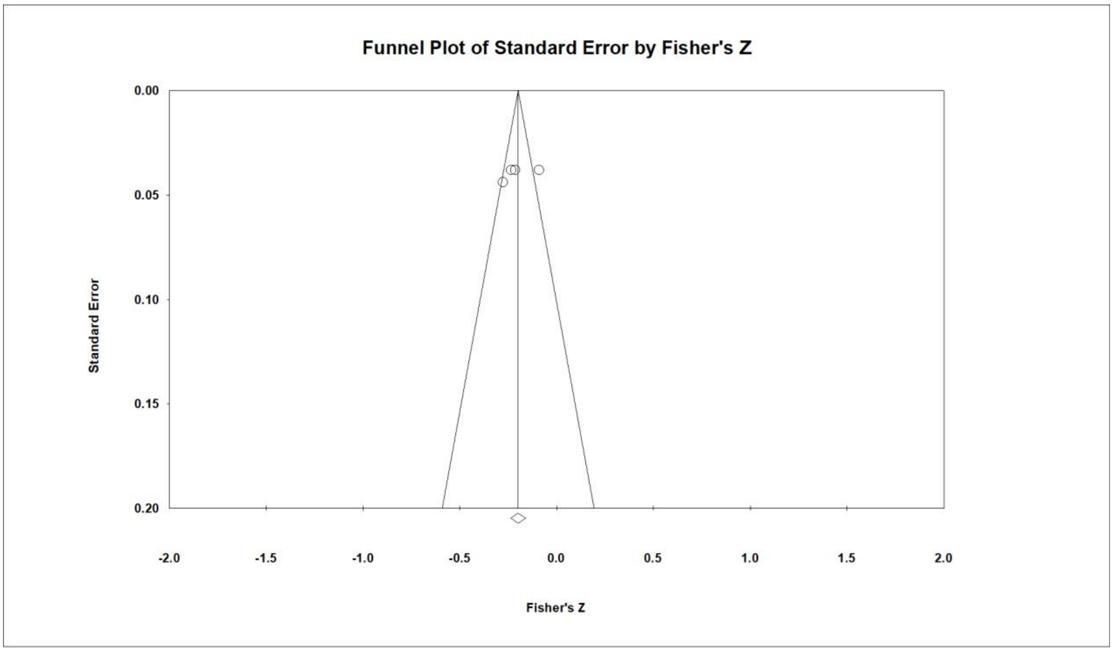

FIGURE 6 **Funnel plot of relation between social support and academic anxiety**
